# Introduction from the Editors

**DOI:** 10.1186/s13223-018-0277-2

**Published:** 2018-09-12

**Authors:** Harold Kim, Richard Warrington, Wade Watson

**Affiliations:** 1 McMaster University, Hamilton, ON Canada; 2 Western University, London, ON Canada; 3 University of Manitoba, Winnipeg, MB Canada; 4 Division of Allergy, Department of Pediatrics, IWK Health Centre, Dalhousie University, Halifax, NS Canada

In 2011, we published a comprehensive supplement entitled, *A Practical Guide for Allergy and Immunology in Canada,* in this journal. The main objective of this supplement was to provide medical students, medical residents, primary-care practitioners and other healthcare professionals with a comprehensive, yet easy-to-follow, series of peer-reviewed articles focussed on all of the common conditions we deal with in the field of allergy and immunology. We believe this objective was achieved since the articles have been downloaded and accessed hundreds of thousands of times. Also, hard copies of the supplement were distributed to thousands of Canadian medical students and residents. Given the high level of readership and interest in these articles over the last 7 years, we felt that it was important to publish updates on the topics addressed in the supplement. In addition, given recent advances in the field of food allergy, we have added an article on early food introduction and have divided the topic of food allergy into two separate papers.

The first article in the supplement provides a basic introduction to the main components and function of the immune system and its role in both health and disease, and serves as a background to the immunopathological disorders discussed in the remainder of this supplement, including asthma, allergic rhinitis, atopic dermatitis (AD), anaphylaxis, food allergy/hypersensitivity, eosinophilic esophagitis (EoE), urticaria, angioedema, drug allergy and primary immunodeficiency disorders (PIDs). Asthma remains the most common chronic respiratory disease in Canada [1], and despite significant improvement in the diagnosis and management of this disorder, the majority of Canadians with asthma remain poorly controlled [2]. In most patients, however, control can be achieved through the use of avoidance measures and appropriate pharmacological interventions. In recent years, three novel biologics have been approved in Canada for the treatment of severe asthma. Allergic rhinitis frequently coexists with asthma [3], and it is often a long-standing condition that goes undetected in the primary-care setting. Over the last few years, two new antihistamines and a novel combination intranasal/antihistamine spray have been added to our treatment armamentarium for allergic rhinitis. AD is one of the most common skin disorders in children [4], and is often the initial step in the “atopic march” (the sequential development of allergic disease manifestations during early childhood), which leads to asthma and/or allergic rhinitis in the majority of afflicted patients [5]. Recently, a new biologic has been added to the treatment options for this condition. Often referred to as “asthma of the esophagus”, EoE is an atopic inflammatory disease of the esophagus that has also become increasingly recognized in children and adults over the last 15–20 years. This supplement provides an overview of the epidemiology and pathophysiology of these common allergic conditions as well as practical strategies for their diagnosis and management.

Allergen-specific immunotherapy is the only potentially disease-modifying therapy for allergic disease and has been proven to be effective for the treatment of allergic rhinitis/conjunctivitis, allergic asthma and stinging insect hypersensitivity. However, despite its proven efficacy, it is frequently underutilized in Canada. In this supplement, the authors review the indications and contraindications, patient selection criteria, and the administration, safety and efficacy of allergen-specific immunotherapy. Importantly, we now have new sublingual immunotherapy products available for the treatment of allergic rhinitis which offer multiple potential benefits over the subcutaneous route, including the comfort of avoiding injections, the convenience of home administration, and a favourable safety profile. It is important to note that we are also on the brink of using food immunotherapy in the treatment of food allergy.

Anaphylaxis is an acute, potentially fatal systemic allergic reaction that requires prompt recognition and treatment; however, both patients and healthcare professionals often fail to recognize and diagnose its early signs and symptoms. The accurate diagnosis and appropriate management of food allergy is also critical since accidental exposure to even minute quantities of the “culprit” food may result in anaphylaxis. The authors discuss the causes, clinical features, diagnosis and management of anaphylaxis and food allergy. In this supplement update, we have divided the topic of food allergy into immunoglobulin E (IgE)-mediated and non-IgE-mediated disease. Importantly, we have had a dramatic change in our approach to early food introduction and, therefore, have added an article on this topic in this update. We now encourage the early introduction of potentially allergenic foods in infancy.

In this supplement, we also review the pathophysiology, diagnosis and treatment of urticaria as well as the work-up and management of isolated angioedema, which varies considerably from that of angioedema that occurs in the presence of urticaria. Although isolated angioedema is often self-limited, laryngeal involvement can lead to fatal asphyxiation in some cases; therefore, prompt recognition and management are imperative. Over the last few years, new treatments for urticaria and angioedema have become available that have expanded our therapeutic options and improved the lives of our patients suffering from these conditions.

Drug allergy encompasses a spectrum of immunologically-mediated hypersensitivity reactions that not only affect patient quality of life, but that may also lead to delayed treatment, unnecessary investigations, and even mortality. In this supplement, the authors examine the most common drug-induced allergic reactions, such allergies to penicillin, sulfonamides, cephalosporins, radiocontrast media, local anesthetics, general anesthetics, acetylsalicylic acid (ASA), non-steroidal anti-inflammatory drugs (NSAIDs) and monoclonal antibodies. The authors also discuss multiple drug hypersensitivity (MDH), a rather novel syndrome characterized by delayed hypersensitivity reactions to two or more structurally unrelated drugs.

This supplement also provides an overview of the major categories of PIDs (a heterogeneous group disorders that result from defects in immune system development and/or function). Although the clinical manifestations of PIDs are highly variable, most disorders involve an increased susceptibility to infection. In fact, many PIDs present as “routine” respiratory infections and, therefore, may go undetected in the primary-care setting. The authors also discuss how the current view of PIDs includes an increasing number of syndromes that are associated with autoimmunity and immune dysregulation as predominant features, rather than an overt pathological risk of infections. This concept of immune dysregulation as “immunodeficiency” is novel and will become increasingly important in the field in the coming years.

For each of the above-mentioned articles, key take-home messages are summarized for quick reference and, where applicable, easy-to-follow flow charts, tables and algorithms are provided to assist clinicians in the identification, diagnosis and treatment of these common allergic diseases. We are confident that readers will not only find this updated supplement educational and informative, but that it will also provide clinicians with a solid base of knowledge and skills in allergy and immunology which they can then incorporate into their respective clinical practices to help improve the care and management of patients with allergic disease. It is also our hope that this supplement will spark further interest in these conditions and in our specialty.

Finally, we would like to thank all of the authors who set aside time from their numerous commitments to write and review these informative articles, the peer reviewers for providing their highly-valued feedback, and the Canadian Society of Asthma and Clinical Immunology (CSACI) and other sponsors who provided the support needed for the development of this important educational initiative.

We sincerely hope you enjoy this updated supplement!

Harold Kim, MD, FRCPC

Richard Warrington, MB, BS, PhD, FRCPC

Wade Watson, MD, MEd, FRCPC

## Abbreviations

AD: atopic dermatitis
EoE: eosinophilic esophagitis
PIDs: primary immunodeficiency disorders
IgE: immunoglobulin E
ASA: acetylsalicylic acid
NSAIDs: non-steroidal anti-inflammatory drugs
MDH: multiple drug hypersensitivity
CSACI: Canadian Society of Asthma and Clinical Immunology
